# Independent and joint associations of neighbourhood greenness and walkability with transportational and recreational physical activity among youth and adults in Canada

**DOI:** 10.1016/j.pmedr.2025.102974

**Published:** 2025-01-16

**Authors:** Natalie Doan, Sebastian A. Srugo, Stephanie A. Prince, Rachel C. Colley, Daniel G. Rainham, Taru Manyanga, Gregory P. Butler, Richard Larouche, Sarah E. Turner, Justin J. Lang

**Affiliations:** a Centre for Surveillance and Applied Research, Health Promotion and Chronic Disease Prevention Branch, Public Health Agency of Canada, Ottawa, ON K1A 0K9, Canada; b School of Public Health Sciences, University of Waterloo, Waterloo, ON N2L 3G1, Canada; c School of Epidemiology and Public Health, Faculty of Medicine, University of Ottawa, Ottawa, ON K1N 6N5, Canada; d Health Analysis Division, Statistics Canada, Ottawa, ON K1A 0T6, Canada; e School of Health and Human Performance, Dalhousie University, Halifax, NS B3H 4R2, Canada; f Division of Medical Sciences, University of Northern British Columbia, Prince George, BC V2N 4Z9, Canada; g Faculty of Health Sciences, University of Lethbridge, Lethbridge, AB T1K 3M4, Canada; h Department of Community Health Sciences, University of Manitoba, Winnipeg, MB R3T 2N2, Canada; i Alliance for Research in Exercise, Nutrition and Activity (ARENA), University of South Australia, Adelaide, SA 5095, Australia

**Keywords:** Neighbourhood greenness, Walkability, Physical activity, Active transportation, Urban health

## Abstract

**Objectives:**

Greener neighbourhoods may support physical activity; however, it is also important to consider whether greener neighbourhoods are walkable. We assessed whether neighbourhood greenness and walkability were independently and jointly associated with transportational (PA_TRA_) and recreational (PA_REC_) physical activity among a nationally representative sample of urban-dwelling youth and adults in Canada.

**Methods:**

We analyzed repeated cross-sectional data from 132,927 urban-dwelling youth (12–17 years) and adults (≥18 years) who participated in the Canadian Community Health Survey (2015–2018). To derive an indicator of neighbourhood greenness, for each survey year, we aggregated mean Normalized Difference Vegetation Index (NDVI) values within 2016 dissemination areas. As a proxy for neighbourhood walkability, we used 2016 Canadian Active Living Environments (Can-ALE) metrics. We used weighted linear regressions to examine whether, and the extent to which, neighbourhood NDVI and the Can-ALE index were independently and jointly associated with self-reported and log-transformed PA_TRA_ and PA_REC_, while adjusting for individual and neighbourhood characteristics.

**Results:**

Among adults, but not youth, higher NDVI was independently associated with higher PA_TRA_ (β = 0.2, 95 % CI 0.02, 0.36) and PA_REC_ (β = 0.3, 95 % CI 0.14, 0.47). Among both youth (β = 0.4, 95 % CI 0.21, 0.66) and adults (β = 0.3, 95 % CI 0.22, 0.35), NDVI and Can-ALE were jointly associated with PA_TRA_.

**Conclusion:**

Living in a neighbourhood that is both greener and more walkable was more strongly associated with higher transportational, but not recreational, physical activity, than either feature alone. These novel findings highlight the importance of designing cities that are both greener and more walkable to promote active living.

## Highlights

• We analyzed data from a nationally representative sample of youth/adults in Canada.
• Greener areas were associated with more physical activity in adults, but not youth.
• Greener and more walkable areas were jointly associated with active transportation.
• Designing neighbourhoods that are greener and more walkable may promote active living.

## 1 Introduction

Physical activity is protective against several chronic diseases and premature mortality (Warburton et al., 2010). Regular participation in physical activity has been linked to reduced risks of developing some types of cancer, osteoporosis, diabetes, and hypertension (Warburton et al., 2010). Despite its protective effects, average levels of physical activity among Canadians consistently fall short of national recommendations (i.e., ≥60 min/day (youth); ≥150 min/week (adults) of moderate-to-vigorous intensity physical activity) based on the Canadian 24-Hour Movement Guidelines (Tremblay et al., 2016). Self-reported data from the 2021 Canadian Community Health Survey (CCHS) showed that 35.0 % of girls, 52.2 % of boys, 50.8 % of women, and 56.4 % of men met these recommendations (Ross et al., 2020; Colley and Saunders, 2023a; Colley and Saunders, 2023b).

Since some features of the built environment are known to be positively associated with physical activity, changing these environmental factors may increase levels of physical activity in the population (Prince et al., 2022a, Prince et al., 2022b; Frumkin et al., 2004; Lawrence et al., 2017; Kärmeniemi et al., 2018). For example, green infrastructure, such as trees and parks, may indirectly benefit health as contexts that support physical activity (Markevych et al., 2017; Maas et al., 2008). However, research on associations between neighbourhood greenness and physical activity has produced mixed results, with some positive associations among studies assessing self-report measures of physical activity (e.g., (McMorris et al., 2015; Thornton et al., 2017)), and some negative/null results (e.g., (Schipperijn et al., 2013; Ord et al., 2013)). These contradictory findings are likely in part influenced by variability in greenness measures (e.g., perceived versus objective, all versus specific forms of greenness [e.g., public parks, gardens]). Still, the role of urban greenness as a health-promoting resource requires further investigation, particularly in terms of specific physical activity domain(s) (e.g., transportational, recreational).

Research suggests that greener neighbourhoods are not always more walkable (Shuvo et al., 2021; Doiron et al., 2020). The extent to which a neighbourhood is walkable is commonly measured by how well a neighbourhood is built to promote active transportation (e.g., points of interests, transit stops, intersections and dwelling density), such as walking (Colley et al., 2019a). In a study conducted in Toronto, Montreal, and Vancouver, researchers found that relatively few areas were both simultaneously characterized as most favourable, or unfavourable, in exposure to greenness, walkability, and air pollution (Doiron et al., 2020). For example, fewer than 2 % of postal codes in these three cities were classified in the highest tertiles for both greenness and walkability, and the lowest tertile for nitrogen oxide (Doiron et al., 2020). In light of this evidence, it is necessary to identify the extent to which the discrepancy between neighbourhood greenness and walkability exists nationally, and investigate whether individuals living in neighbourhoods that are both green and walkable are more physically active than individuals living in neighbourhoods that are only green, only walkable or neither.

To address these research gaps, we aimed to identify whether neighbourhood greenness interacts with walkability in its association with self-reported physical activity (transportational, recreational) among a large and nationally representative sample of youth and adults living in Canadian urban areas. We conducted four secondary analyses to assess for heterogeneity in the primary associations by sex, age group, and community size and to test the robustness of our primary models.

## 2 Methods

### 2.1 Data sources and study sample

We used data from four cycles of the Canadian Community Health Survey (CCHS), collected annually from 2015 to 2018. The CCHS is an ongoing, repeated cross-sectional, nationally representative survey that uses a two-stage stratified cluster design to collect self-reported health information from individuals aged 12 years and over in Canada (response rate: 2015–16 = 59.5 %, 2017–18 = 61.5 %) (Statistics Canada, 2017a; Statistics Canada, 2024a). Excluded from the CCHS are persons living on Indigenous reserves and settlements, individuals who are institutionalized, and full-time members of the Canadian Forces (Statistics Canada, 2017a; Statistics Canada, 2024a). Prior to data collection, Statistics Canada obtained informed consent from all participants. This research is exempt from Research Ethics Board review as it relies on publicly available anonymized data (Canadian Institutes of Health Research, 2022). We analyzed data from the 2015–16 and 2017–18 combined CCHS datafiles because physical activity data were collected in all provinces and territories. For this study, we focused on individuals living in urban areas (population centres ≥1000 persons and ≥400/km^2^) (Statistics Canada, 2017b).

Environmental data were supplied by the Canadian Urban Environmental Health Research Consortium (CANUE) and Statistics Canada, and were assigned to participants based on the dissemination areas of their place of residence–relatively small and stable geographic units that typically encompass a population of 400–700 people. Since the 2016 vintage of the dissemination areas best aligned with the survey data, we assigned area-level data to participants based on the 2016 dissemination area corresponding to their residential postal code. The Postal Code Conversion File Plus (PCCF+) (Statistics Canada, 2024b) was employed to assign 2016 dissemination area to these participants. **Supplemental Table 1** presents the linkages performed.

Among the individuals living in urban areas (*n* = 153,708), we excluded individuals with missing primary and covariate data (13.5 %), resulting in an analytic sample of 132,927 individuals.

### 2.2 Measures

#### 2.2.1 Neighbourhood greenness

We merged annual Landsat 8 Normalized Difference Vegetation Index (NDVI) data (Google, 2024a; Google, 2024b) and the DMTI postal code file (DMTI Spatial Inc, 2015), then spatially joined Statistics Canada's 2016 Cartographic Census Boundary files to obtain the latitude, longitude, and 2016 dissemination area for each postal code (Statistics Canada, 2019). To generate a measure of neighbourhood greenness for each 2016 dissemination area (each year), we aggregated annual mean NDVI values (postal codes) within the 2016 dissemintation area. NDVI values range from −1 (no greenness) to 1 (all green); however, similar to previous research (Crouse et al., 2017), we excluded negative values (NDVI<0.00) because they may indicate reflective surfaces (e.g., water, ice). We chose to use Landsat NDVI data because it provides high-quality spatial resolution (30 m × 30 m) and is the most widely used satellite-derived indicator of the quantity of green vegetation. For this study, we focused on continuous NDVI scores, but also derived a categorical greenness quartile variable to cross-tabulate with walkability (1 = neighbourhoods with the lowest greenness, 4 = neighbourhoods with the highest greenness).

#### 2.2.2 Neighbourhood walkability

Walkability is the extent to which the community is designed to encourage walking (Frank et al., 2010). Key to the concept of walkability is whether an area is conducive to utilitarian walking (i.e., walking to destinations, commuting to work/school, running errands) (Colley et al., 2019a). Therefore, as a proxy for neighbourhood walkability, we used a metric that was designed to assess the degree to which neighbourhood infrastructure promotes active transportation (Herrmann et al., 2019) – the Canadian Active Living Environments (Can-ALE) (Herrmann et al., 2019). Can-ALE values are based on measures of residential density, street connectivity (roads, footpaths), destinations of interest (amenities), and transit stops (Herrmann et al., 2019). Since Can-ALE metrics were derived for census years, and the versions are not comparable, we assigned the 2016 version since it is most temporally aligned with the year of data collection among CCHS cycles included in the analysis. We primarily used the continuous Can-ALE index, but also used the modified categorical Can-ALE class metric to cross-tabulate with greenness. The Can-ALE class metric characterizes the favourability of the active living environments on a scale from 1 (very low) to 5 (very high) (Herrmann et al., 2019); however, since very few individuals lived in Can-ALE classes 4 and 5, we collapsed these two classes into one category which we refer to as “highly walkable”.

#### 2.2.3 Physical activity

To characterize the physical activity of respondents, we used data from the Physical Activity for Youth (PAY) and the Physical Activity for Adults (PAA) modules (see **Appendices A** and **B**). As part of the CCHS household interview, respondents were asked to provide an estimate of the time spent in the last seven days engaged in various physical activity domains. For this study, we focused on two domains: transportational (PA_TRA_) and recreational (PA_REC_) physical activity. Similar to previous research (Colley et al., 2018; Colley et al., 2019b), average daily values for each physical activity domain were calculated by dividing the weekly total for each domain by seven. To account for negative skewness of self-reported physical activity data, before statistical modelling, the physical activity variables were log-transformed using a natural logarithm with a constant of one.

#### 2.2.4 Covariates

As part of the CCHS interview, sociodemographic, and health information data were collected at the participant and household levels. Several covariate measures were derived, including self-reported sex (male, female), age (years), Indigenous identity and race/ethnicity (Black, East Asian [Chinese, Korean, Japanese], Indigenous [First Nations, Inuk/Inuit, Métis], Latin American [Arab, West Asian], Middle Eastern, South Asian, Southeast Asian [Southeast Asian, Filipino], White, and another race/ethnicity [individuals who selected “Other” or more than one response that did not fall exclusively within our prespecified race/ethnicity categories]), immigration (Canadian-born, ≥10 years since immigration, <10 years since immigration, temporary resident), household educational attainment (some secondary school or less, secondary school graduate, some post-secondary or more), home ownership (own, rent), and self-rated general health status (excellent/very good/good, fair/poor). Additionally, we derived a variable that reflects the season based on the interview month (spring [March–May], summer [June–August], fall [September–November], winter [December–February]) and community size (small population centre [1000-29,999], medium population centre [30,000-99,999], larger urban population centre [≥100,000]). To obtain measures of neighbourhood marginalization, we used the factor scores for each of the four dimensions of the 2016 Canadian Marginalization Index (CAN-Marg): households and dwellings, material resources, age and labour force, immigration and visible minority (Matheson et al., 2021).

### 2.3 Statistical analyses

To characterize the study population, we produced descriptive statistics, including unweighted frequencies, weighted proportions and means. We applied Statistics Canada's sampling variability guidelines to indicate the reliability of proportion estimates using the coefficient of variance (sampling error relative to the sample size) (Statistics Canada, 2017a; Statistics Canada, 2024a).

For our primary analyses, we assessed the unweighted Pearson's correlation between NDVI scores and the Can-ALE index to determine the extent to which continuous neighbourhood greenness and walkability were correlated. To assess the degree of overlap between the categorical greenness quartiles and modified Can-ALE class variable, we used weighted chi-square analyses. We employed weighted multivariable linear regression models to examine whether neighbourhood greenness (NDVI scores) and walkability (Can-ALE index) were associated with PA_TRA_ and PA_REC_. When modelling each physical activity measure, main effect and interaction models were tested. In this study, we use the term “effect” to refer to statistical associations and not to imply causality. In each main effect model, NDVI scores and the Can-ALE index were included as individual main effects along with covariates. For interaction models, we added a two-way interaction term (NDVI × Can-ALE index) to assess joint associations.

As secondary analyses to determine the robustness of our primary results, we ran four sensitivity analyses. First, to identify potential selection bias, we assessed the overlap between the weighted proportion and mean estimates 95 % CI to gauge whether the individuals included in the analytic sample differed from those excluded due to missing data. Second, using a reduced sample of youth only, we developed stratified models to examine variations in the associations of interest across sex (male, female) and community size (small, medium, large population centres). Third, using a reduced sample of adults only, we ran the same stratified models as with the youth data, with the addition of an age group stratification (adults only; 18–39, 40–64, 65+ years). Last, since outdoor vegetation and grounds may be covered by snow in the winter, we conducted a sensitivity analysis to assess whether the results were consistent when we removed individuals who participated in the CCHS during the winter months (December–February).

To facilitate interpreting significant main effects and interactions, we assessed the direction and (relative) magnitude of the regression (β) estimates.

Since we pooled the 2015–16 and 2017–18 CCHS combined files, we divided the weights by two. The sampling weights facilitated the generation of estimates representative of the Canadian population, and the replicate bootstrap weights allowed us to calculate 95 % confidence intervals (CI) that account for the complex survey design and non-response bias. Since there were differences in the PAY and PAA modules, we present the results for youth (12–17 years) and adults (≥18 years), separately. All analyses were conducted using SAS Enterprise Guide (SAS Institute Inc, 2024) and RStudio (R Core Team, 2020), and weighted using Statistics Canada's survey and bootstrap weights.

## 3 Results

### 3.1 Weighted descriptive statistics

Table 1 presents the weighted descriptive statistics. On average, individuals lived in neighbourhoods low in greenness (0.3, 95 % CI: 0.3, 0.3 for both youth and adults). The Can-ALE class distribution suggests that a lower proportion of youth (12.4 %, 95 % CI: 11.4, 13.4) lived in highly walkable neighbourhoods compared to adults (18.6 %, 95 % CI: 17.7, 19.4). Youth reported a daily average of 30.4 min (95 % CI: 29.0, 31.8) of PA_TRA_ and 35.6 (95 % CI: 34.3, 36.8) minutes of PA_REC_, while adults reported a daily average of 16.2 (95 % CI: 15.8, 16.6) and 16.2 (95 % CI: 15.9, 16.5) minutes of PA_TRA_ and PA_REC_, respectively.

**Table 1:** Descriptive statistics of urban-dwelling youth and adults in the 2015–2018 Canadian Community Health Survey (*n* = 132,927).

Characteristics	Youth *n* = 8825 (unweighted) n = 1,381,6560 (weighted)		Adults n = 124,102 (unweighted) *n* = 20,935,853 (weighted)	
	Sample size	% or mean (95 % CI)	Sample size	% or mean (95 % CI)
*Individual-level*				
Sex				
Male	4619	52.7 (51.8, 53.5)	55,818	48.9 (48.7, 49.1)
Female	4206	47.3 (46.5, 48.2)	68,284	51.0 (50.9, 51.3)
Mean age, years	–	14.5 (14.5, 14.6)	–	47.0 (46.9, 47.1)
Household educational attainment				
Some high school or less	244	2.9 (2.4, 3.4)	10,904	5.0 (4.8, 5.3)
High school graduate	880	10.7 (9.8, 11.7)	20,225	13.5 (13.2, 13.9)
Some post-secondary or more	7701	86.4 (85.3, 87.4)	92,973	81.5 (81.0, 81.9)
Indigenous identity and race/ethnicity				
Black	274	4.9 (4.1, 5.6)	2237	2.9 (2.7, 3.2)
East Asian (Chinese, Japanese, Korean)	315	4.7 (4.1, 5.4)	4245	5.6 (5.2, 5.9)
Indigenous (First Nations, Inuk/Inuit, Métis)	628	5.1 (4.5, 5.6)	5423	3.2 (3.1, 3.4)
Latin American	98	1.5 (1.1, 1.9)	1219	1.6 (1.4, 1.7)
Middle Eastern (Arab, West Asian)	225	3.9 (3.3, 4.5)	1596	2.4 (2.2, 2.6)
South Asian	382	7.1 (6.3, 8.0)	3376	5.6 (5.3, 5.9)
Southeast Asian (Southeast Asian, Filipino)	336	4.7 (4.0, 5.4)	2449	3.7 (3.4, 3.9)
White	6050	60.5 (59.1, 61.9)	100,333	71.1 (70.4, 71.7)
Another race/ethnicity (multiple origins, Other)	517	7.6 (6.8, 8.4)	3224	4.0 (3.8, 4.2)
Immigration status				
Canadian-born	7699	83.6 (82.5, 84.7)	98,054	68.9 (68.3, 69.5)
≥ 10 years since immigration	368	5.6 (4.8, 6.3)	17,833	20.6 (20.1, 21.1)
<10 years since immigration	633	8.9 (8.0, 9.8)	5778	7.5 (7.2, 7.8)
Temporary resident	125	1.9 (1.5, 2.4)	2437	3.0 (2.8, 3.2)
Home ownership				
Own	7153	77.5 (76.1, 78.8)	83,471	69.3 (68.6, 69.9)
Rent	1672	22.5 (21.2, 23.9)	40,631	30.7 (30.1, 31.4)
Self-rated general health				
Fair/poor	311	3.7 (3.2, 4.3)	17,071	11.1 (10.8, 11.4)
Excellent/very good/good	8514	96.3 (95.7, 96.8)	107,031	88.6 (88.6, 89.2)
Season				
Spring	2289	25.6 (24.2, 27.0)	31,383	25.2 (24.8, 25.7)
Summer	1851	21.5 (20.2, 22.9)	29,727	23.5 (23.1, 23.9)
Fall	2222	25.1 (23.7, 26.5)	31,507	25.8 (25.4, 26.3)
Winter	2463	27.8 (26.4, 29.3)	31,485	25.5 (25.0, 25.9)
Community size				
Small population centre (1000–29,999 persons)	2227	12.7 (12.0, 13.4)	30,241	12.0 (11.5, 12.5)
Medium population centre (30,000–99,999 persons)	1455	10.6 (9.9, 11.2)	22,841	11.3 (10.8, 11.8)
Large population centre (≥100,000 persons)	5143	76.7 (75.8, 77.6)	71,020	76.7 (76.2, 77.3)
Mean PA_TRA_, minutes/day^1^	–	30.4 (29.0, 31.8)	–	16.2 (15.8, 16.6)
Mean PA_REC_, minutes/day^1^	–	35.6 (34.3, 36.8)	–	16.2 (15.9, 16.5)
Year of data collection				
2015	2155	24.8 (24.1, 25.5)	28,606	23.8 (23.6, 24.0)
2016	2358	25.2 (24.5, 25.9)	31,948	24.9 (24.7, 25.1)
2017	2309	25.7 (24.9, 26.4)	32,740	25.5 (25.3, 25.7)
2018	2003	24.4 (23.6, 25.1)	30,808	25.8 (25.6, 26.0)
Area-level				
CAN-Marg scores				0.2 (0.2, 0.2)
Mean households and dwelling, score^2^	–	−0.2 (−0.2, −0.2)	–	
Mean material resources, score^2^	–	−0.2 (−0.2, −0.2)	–	−0.2 (−0.2, −0.2)
Mean age and labour force, score^2^	–	−0.4 (−0.4, −0.3)	–	−0.2 (−0.2, −0.2)
Mean immigration and visible minority, score^2^	–	0.5 (0.5, 0.6)	–	0.4 (0.4, 0.4)
Mean Can-ALE index	–	0.4 (0.3, 0.5)	–	1.0 (0.9, 1.1)
Modified Can-ALE class^3^				
Class 1	2355	16.8 (15.9, 17.7)	28,269	14.1 (13.4, 14.8)
Class 2	3926	40.0 (38.7, 41.3)	51,553	37.5 (36.4, 38.7)
Class 3	1978	30.8 (29.4, 32.1)	30,843	29.8 (28.5, 31.1)
Class 4/5	566	12.4 (11.4, 13.4)	13,437	18.6 (17.7, 19.4)
Mean, Normalized Difference Vegetation Index (NDVI)	–	0.3 (0.3, 0.3)	–	0.3 (0.3, 0.3)

*Notes:* Data are from urban individuals who participated in the 2015–2018 Canadian Community Health Survey – Annual component (*n* = 132,927). Presented are the unweighted n and weighted % (95 % confidence interval), unless otherwise specified. Acronyms: PA_TRA_ = transportational physical activity; PA_REC_ = recreational physical activity; CAN-Marg = Canadian Marginalization Index; Can-ALE = Canadian Active Living Environments.

1 Daily average (minutes/day) domain-specific physical activity was calculated by dividing the weekly domain-specific physical activity by 7.

2 Factor scores for each of the 2016 CAN-Marg dimensions is on an asymmetrical standardized scale, with a mean of zero and standard deviation.

3 The Can-ALE index is the sum of the z-scores for each ALE measure (intersection density, dwelling density, points of interest, and transit measures).

Characteristics of individuals who were included and excluded from the primary analytic sample are compared in **Supplemental Table 2**. These samples differed by various sociodemographic and neighbourhood level characteristics; however, most differences were trivial. Notably, included individuals were more likely to live in more walkable areas than excluded individuals whereas they lived in equally green areas.

### 3.2 Primary results

#### 3.2.1 Bivariate analyses

NDVI scores and the Can-ALE index were inversely and weakly correlated for youth (*r* = −0.1, *p* < 0.0001) and adults (*r* = −0.3, p < 0.0001). Consistent with our correlation analyses using the continuous NDVI and Can-ALE variables, weighted cross-tabulations of categorical variables showed that as neighbourhood greenness moved from the lowest to highest quartiles, neighbourhoods were less likely to be highly walkable (Table 2).

**Table 2:** Cross-tabulation, sample size and percent, of neighbourhood greenness quintiles and walkability classes among youth and adults in the 2015–2018 Canadian Community Health Survey (*n* = 132,927).

Youth (unweighted *n* = 8825)					
		Can-ALE, n(%)			

		1	2	3	4/5

NDVI, n(%)	1	697 (3.8)	833 (8.6)	421 (7.0)	234 (5.7 %)
	2	364 (2.9)	902 (10.0)	548 (9.1)	156 (2.9 %)
	3	402 (3.4)	1065 (10.7)	543 (8.2)	121 (2.8 %)
	4	892 (6.7)	1126 (10.8)	466 (6.5)	55 (1.1 %)

Adults (unweighted *n* = 124,102)					
		Can-ALE, n(%)			

		1	2	3	4/5

NDVI, n(%)	1	7518 (3.0)	10,261 (6.8)	6905 (6.1)	6661 (9.1)
	2	5139 (2.7)	12,075 (9.0)	8339 (8.5)	3558 (4.8)
	3	5524 (2.9)	14,522 (10.6)	8362 (8.4)	2284 (3.1)
	4	10,088 (5.5)	14,695 (11.1)	7237 (6.9)	934 (1.5)

*Notes:* Data are from urban individuals who participated in the 2015–2018 Canadian Community Health Survey – Annual component (n = 132,927). Acronyms: NDVI = Normalized Difference Vegetation Index; Can-ALE = Canadian Active Living Environments. Presented are the unweighted n (weighted %).

#### 3.2.2 Main effect and interaction models

Table 3 presents the primary results for youth and adults. The main effect models revealed that neighbourhood NDVI was neither independently associated with PA_TRA_ (β = 0.5, 95 % CI: −0.06, 0.99) nor PA_REC_ (β = −0.1, 95 % CI: −0.69, 0.49) among youth. However, higher neighbourhood NDVI was independently associated with higher PA_TRA_ (β = 0.2, 95 % CI: 0.02, 0.36) and PA_REC_ (β = 0.3, 95 % CI: 0.14, 0.47) among adults. Among both youth and adults, higher neighbourhood Can-ALE was independently associated with higher PA_TRA_ (youth: β = 0.1, 95 % CI: 0.03, 0.10; adults: β = 0.1, 95 % CI: 0.06, 0.08), but not PA_REC_ (youth: β = 0.0, 95 % CI: −0.02, 0.05; adults: β = 0.0, 95 % CI: 0.00, 0.01). For both youth and adults, the interaction between neighbourhood NDVI and Can-ALE was significantly associated with PA_TRA_ (youth: β = 0.4, 95 % CI: 0.21, 0.66; adults: β = 0.3, 95 % CI: 0.22, 0.35), but not PA_REC_ (youth: β = −0.1, 95 % CI: −0.34, 0.16, adults: β = 0.0, 95 % CI: −0.01, 0.07).

**Table 3:** Primary analyses testing the main effects and interactions, reported as β (95 % CI), between neighbourhood greenness and walkability in relation to physical activity among youth and adults in the 2015–2018 Canadian Community Health Survey (n = 132,927).

Models	Transportational physical activity^1^	Recreational physical activity^1^
	β (95 % CI)	β (95 % CI)
*Youth (n = 8825)*		
Main effects		
NDVI	0.5 (−0.06, 0.99)	−0.1 (−0.69, 0.49)
Can-ALE	0.1 (0.03, 0.10)	0.0 (−0.02, 0.05)
Interaction		
NDVI × Can-ALE	0.4 (0.21, 0.66)	−0.1 (−0.34, 0.16)
*Adults (n* *= 124,102)*		
Main effects		
NDVI	0.2 (0.02, 0.36)	0.3 (0.14, 0.47)
Can-ALE	0.1 (0.06, 0.08)	0.0 (0.00, 0.01)
Interaction		
NDVI × Can-ALE	0.3 (0.22, 0.35)	0.0 (−0.01, 0.07)

*Notes:* Data are from urban individuals who participated in the 2015–2018 Canadian Community Health Survey – Annual component (n = 132,927). β and 95 % CI are rounded to one and two decimal places, respectively. Estimates are adjusted for sex, age, Indigenous identity and race/ethnicity, immigration status, educational attainment, home ownership, self-rated general health, season of data collection, community size, neighbourhood households and dwelling, neighbourhood material resources, neighbourhood age and labour force, and neighbourhood immigration and visible minority. Acronyms: β = beta coefficient, CI = confidence interval; NDVI = Normalized Difference Vegetation Index; Can-ALE = Canadian Active Living Environments.

1 Daily average (minutes/day) domain-specific physical activity was calculated by dividing the weekly domain-specific physical activity by 7, and then log-transforming with a natural algorithm.

### 3.3 Secondary models

The findings from the sex-stratified analyses indicate that the interaction between neighbourhood NDVI and Can-ALE was not significantly associated with PA_TRA_ among female youth (β = 0.4, 95 % CI: −0.01, 0.77, **Supplemental Table 3**), and neighbourhood NDVI was not independently associated with PA_TRA_ among adult males (β = 0.1, 95 % CI: −0.09, 0.38, **Supplemental Table 4**).

When the adult sample was stratified by age group, neighbourhood NDVI was no longer independently associated with PA_TRA_ among any age group (18–29 years: β = 0.2, 95 % CI: −0.05, 0.52; 40–64 years: β = 0.2, 95 % CI: −0.10, 0.43; 65+ years: β = −0.1, 95 % CI: −0.42, 0.15), or with PA_REC_ among adults 18–39 years (β = 0.1, 95 % CI: −0.18, 0.38). The interaction between neighbourhood NDVI and Can-ALE was not significantly associated with PA_TRA_ among adults aged 65+ years (β = 0.1, 95 % CI: −0.02, 0.16).

The analyses stratified by population centre size revealed that neighbourhood NDVI was typically more strongly associated with PA_TRA_ and PA_REC_ among adults living in larger population centres. Moreover, the interaction between neighbourhood NDVI and Can-ALE in relation to PA_TRA_ was not significant among adults living in small (β = −0.1, 95 % CI: −0.43, 0.31) or medium (β = 0.3, 95 % CI: 0.00, 0.61) population centres.

The results of our analyses excluding individuals who participated in the CCHS in the winter months (**Supplemental Table 5**) were consistent with the primary analyses, with the exception that neighbourhood NDVI was no longer independently associated with PA_TRA_ among adults (β = 0.1, 95 %: CI -0.04, 0.34).

## 4 Discussion

Among a nationally representative sample of urban-dwelling youth and adults in Canada, we found that living in a neighbourhood higher in greenness was associated with higher transportational and recreational physical activity for adults, but not for youth. Associations between living in a greener neighbourhood and transportational, but not recreational, physical activity were stronger for those living in more walkable neighbourhoods regardless of age.

Among adults, our findings align with existing evidence suggesting that neighbourhood greenness may promote physical activity by providing attractive opportunities for adults to walk, run, cycle, and engage in other physical activities, whether for commuting or leisure. Features such as greenery, parks, landscaping, and shade can enhance the appeal of using active modes of transportation (Pikora et al., 2003; Sallis et al., 1997). Similarly, greenness can provide adults with opportunities to engage in outdoor activities, including walking, running, playing sports, and gardening (Rodriguez-Villamizar et al., 2024). Since youth may rely more heavily on useable green areas (greenspaces) for physical activity, regardless of greenness, than adults (Almanza et al., 2012), it is not surprising that we did not find significant associations between neighbourhood greenness (absolute amount of all types of vegetation) and physical activity in youth.

Among both youth and adults, our study revealed that neighbourhood greenness and walkability were jointly associated with transportational, but not with recreational physical activity. These findings corroborate the body of evidence that individuals may be more inclined to undertake physical activity as a mode of transportation in greener neighbourhoods when these neighbourhoods are also supportive of walking (e.g., (Hawthorne, 1989; James et al., 2017)). Furthermore, while neighbourhood greenness and walkability may facilitate walking for leisure, certain types of neighbourhood infrastructure may play a more critical role in shaping active transportation than other forms of physical activity (e.g., (Wendel-Vos et al., 2007; Saelens and Handy, 2008)). Empirical evidence supports this explanation as particular neighbourhood infrastructure features (e.g. sidewalks, multi-use paths) are associated with utilitarian walking more than other types of physical activity, including recreational walking (e.g., (McCormack, 2017; Farkas et al., 2019)). These findings extend previous research by highlighting neighbourhood greenness and walkability may interact to shape domain- and type-specific physical activity.

Age-group stratified analyses revealed stronger joint associations between neighbourhood greenness and walkability with active transportation among adults 18–39 and 40–64 years, compared to adults 65+ years. This age-related difference in the associations may reflect a greater use of active transportation and multi-modal travel (e.g., walking and public transit) among younger, compared to older adults in industrialized countries (Davis et al., 2012; Borhani et al., 2024). Furthermore, adults aged 65+ years are more likely to have health-related conditions that limit mobility, which may explain why associations between neighbourhood greenness and physical activity were not significant among this group. Corroborating existing evidence (McMorris et al., 2015), these findings underscore the importance of adopting a life course perspective when investigating associations between built environments and physical activity to identify heterogeneity in the built environment- physical activity associations across various life stages (Prince et al., 2022a, Prince et al., 2022b).

For both independent and joint associations, we observed a gradient in the significance and strength of associations according to population centre size – associations were more pronounced among larger population centres. These findings extend previous Canadian research that found stronger associations between neighbourhood walkability (using Walk Score®) and physical activity in larger (versus smaller) population centres (Thielman et al., 2015). Since our measure of walkability is based on features such as dwelling and intersection density (including off-road footpaths and trails), integrating these design features, along with greenness, may have significant benefits for residents living in larger population centres (e.g., larger suburbs, cities). Given that dense urban areas are more likely to have these features, but are less likely to be highly green—as demonstrated by our estimates of neighbourhoods that are in the highest Can-ALE and NDVI categories (∼1 %)—increasing access to greenness in urban areas may be associated with increased physical activity. Current national initiatives, such as creating new national parks in urban areas and tree planting initiatives (Parks Canada, 2024; Parks Canada, 2021; Government of Canada, 2024), may play a critical role in promoting human and planetary health through increasing biodiversity and air quality, and mitigating harms associated with climate change (Sadler et al., 2010).

### 4.1 Strengths and limitations

To the best of our knowledge, this is the first study to investigate whether neighbourhood greenness and walkability were independently and jointly associated with transportational and recreational physical activity among urban-dwelling youth and adults in Canada. By leveraging data from the CCHS and CANUE, we were able to assess these associations among a large and geographically diverse population-based sample of youth and adults living in Canada. This large sample size afforded us the ability to identify differences according to sex, age group, and population centre size, and adjust for many important potentially confounding individual and neighbourhood level factors.

Our study had several limitations that should be considered when interpreting the results. First, the cross-sectional design of the CCHS limits our ability to assess the temporality of the associations between features of the neighbourhood environment and physical activity. Second, while the CCHS datasets are nationally-representative of individuals in the ten provinces and three territories in important ways, the weighted estimates are not representative of individuals excluded from the sampling frame (as described above) and do not correct for all forms of under-representation. Third, while NDVI values from Landsat satellite imagery are objective and available countrywide, they do not allow to differentiate between types of vegetation and evaluate factors such as greenness useability, accessibility, and quality. This is an important area for further research. Additionally, while Can-ALE incorporates high-quality and open-source data, metrics may overestimate walkability in larger population centres because active living environments were assessed based on indicators that are more common in urban areas (e.g., residential density). Fourth, we relied on self-report questionnaires to assess domain-specific PA, which are subject to social desirability and recall biases (Colley et al., 2018). Lastly, although assigning area-level measures based on the dissemination areas corresponding to participants' residential postal codes allowed us to characterize neighbourhood greenness and walkability, it does not capture exposure from other spatially relevant contexts (e.g., work, school, routes travelled).

## 5 Conclusion

In conclusion, we found that higher neighbourhood greenness and walkability were independently associated with transportational and recreational physical activity among adults, and jointly associated with transportational physical activity among both youth and adults. The age-specific findings emphasize the need for additional research that focuses on the associations between specific aspects of neighbourhood green infrastructure (types, quality, accessibility) on the physical activity of specific age groups. Nevertheless, our study underscores the importance of designing urban neighbourhoods that are both greener and conducive to active living.

## Funding sources

This research did not receive any specific grant from funding agencies in the public, commercial, or not-for-profit sectors. Open access publication of this paper is supported by the 10.13039/100011094Public Health Agency of Canada.

## CRediT authorship contribution statement

**Natalie Doan:** Writing – review & editing, Writing – original draft, Methodology, Formal analysis, Data curation, Conceptualization. **Sebastian A. Srugo:** Writing – review & editing, Methodology, Conceptualization. **Stephanie A. Prince:** Writing – review & editing, Methodology, Conceptualization. **Rachel C. Colley:** Writing – review & editing, Methodology, Conceptualization. **Daniel G. Rainham:** Writing – review & editing, Methodology. **Taru Manyanga:** Writing – review & editing. **Gregory P. Butler:** Writing – review & editing, Conceptualization. **Richard Larouche:** Writing – review & editing. **Sarah E. Turner:** Writing – review & editing. **Justin J. Lang:** Writing – review & editing, Supervision, Methodology, Conceptualization.

## Declaration of competing interest

The authors declare that they have no known competing financial interests or personal relationships that could have appeared to influence the work reported in this paper.

## Data Availability

The data that has been used is confidential.

## Appendix A Supplementary data

Appendix A: The Canadian Community Health Survey items used to assess transportational and recreational physical activity among youth. Appendix B: The Canadian Community Health Survey items used to assess transportational and recreational physical activity among adults. Image 1
