# Supplementary material for: Independent and joint associations of neighbourhood greenness and walkability with transportational and recreational physical activity among youth and adults in Canada
Source: Prev Med Rep. 2025 Jan 16;50:102974. doi: 10.1016/j.pmedr.2025.102974 (PMC11803170; doi:10.1016/j.pmedr.2025.102974)
Supplement: Supplementary file 1 — Appendix A: The Canadian Community Health Survey items used to assess transportational and recreational physical activity among youth. Appendix B: The Canadian Community Health Survey items used to assess transportational and recreational physical activity among adults. [file mmc1.docx]

**Appendix A – Physical Activity Youth module**

The Canadian Community Health Survey items used to assess transportational and recreational physical activity among youth.

**Transportational physical activity (PA_TRA_)**

Variable name: PAYDVTTR – Total minutes of active transportation - last 7 days

- In the last seven days, did you use active ways like walking or cycling to get to places such as [school, the bus stop, the shopping centre work/ school] or to visit friends? *Do not include walking, cycling or other activities done purely for leisure. These activities will be asked about later.*
- How much time did you spend using active ways to get to places?

**Recreational physical activity (PA_REC_)**

Variable name: PAYDVADL – Total minutes of physical activities during leisure - last 7 days

- In the last seven days, did you do physical activities in your leisure time, including exercising, playing an organized or non-organized sport or playing with your friends?
- Did any of these recreational physical activities make you sweat at least a little and breathe harder?
- How much time did you spend doing these leisure-time activities that made you sweat at least a little and breathe harder?

**Appendix B – Physical Activity Adult module**

The Canadian Community Health Survey items used to assess transportational and recreational physical activity among adults.

**Transportational physical activity (PA_TRA_)**

Variable name: PAADVTRV – Active transportation minutes per week

- In the last seven days, did you use active ways like walking or cycling to get to places such as work, school, the bus stop, the shopping centre or to visit friends? *Do not include walking, cycling or other activities done purely for leisure. These activities will be asked about later.*
- In the last seven days, on which days did you do these activities?
- How much time in total, in the last seven days, did you spend doing these activities? Please only include activities that lasted a minimum of 10 continuous minutes

**Recreational physical activity (PA_REC_)**

Variable name: PAADVREC – Recreational activity minutes per week

- Not including activities you just reported, in the last seven days, did you do sports, fitness or recreational physical activities, organized or non-organized, that lasted a minimum of 10 continuous minutes? Interviewer’s note: Examples are walking, home or gym exercise, swimming, cycling, running, skiing, dancing and all team sports.
- Did any of these recreational physical activities make you sweat at least a little and breathe harder?
- In the last seven days, on which days did you do these recreational activities that made you sweat at least a little and breathe harder?
- In the last seven days, how much time in total did you spend doing these activities that made you sweat at least a little and breathe harder?

**APPENDIX C – SUPPLEMENTAL TABLES**

**Supplemental Table 1.** Year of the area-based indicators (NDVI, Can-ALE, CAN-Marg) that were linked to each cycle of the Canadian Community Health Survey, 2015-2018.

| CCHS Cycle | Dissemination area^1^ | NDVI | Can-ALE | CAN-Marg |
| --- | --- | --- | --- | --- |
| 2015 | 2016 | 2015 | 2016 | 2016 |
| 2016 | 2016 | 2016 | 2016 | 2016 |
| 2017 | 2016 | 2017 | 2016 | 2016 |
| 2018 | 2016 | 2018 | 2016 | 2016 |

*Notes:* CCHS = Canadian Community Health Survey; NDVI = Normalized Difference Vegetation Index; Can-ALE = Canadian Active Living Environment; CAN-Marg = Canadian Marginalization Index.

^1^ Dissemination areas were assigned to participants using the Postal Code Conversion File Plus (PCCF+), which uses a probabilistic method to match postal codes to census geographic areas. We used the PCCF+ to assign 2016 DAs to participants in the 2015-16 CCHS; Statistics Canada used the PCCF+ to assign 2016 DAs to participants in the 2017-18 CCHS.

**Supplemental Table 2.** Secondary analysis comparing the weighted descriptives statistics for urban individuals included (complete cases) and excluded from the analyses (due to missing data) among youth and adults in the 2015-2018 Canadian Community Health Survey (n=153,708).

| Characteristics | Youth | | Adults | |
| --- | --- | --- | --- | --- |
|  | Complete cases  % or mean  (95%CI)  n=8,825 | Excluded cases  % or mean  (95%CI)  n=3,020 | Complete cases  % or mean  (95%CI)  n=124,102 | Excluded cases  % or mean  (95%CI)  n=17,761 |
| Individual-level |  |  |  |  |
| Sex |  |  |  |  |
| Male | 52.7 (51.8, 53.5) | 47.8 (45.5, 50.2) | 48.9 (48.7, 49.1) | 49.8 (48.5, 51.1) |
| Female | 47.3 (46.5, 48.2) | 52.2 (49.8, 54.5) | 51.1 (50.9, 51.3) | 50.2 (48.9, 51.5) |
| Mean age, years | 14.5 (14.5, 14.6) | 14.3 (14.2, 14.4) | 47.0 (46.9, 47.1) | 49.7 (49.1, 50.2) |
| Indigenous identity and race/ethnicity |  |  |  |  |
| Black | 4.9 (4.1, 5.6) | 5.4 (3.8, 6.9) | 2.9 (2.7, 3.2) | 3.9 (3.2, 4.7) |
| East Asian (Chinese, Japanese, Korean) | 4.7 (4.1, 5.4) | 4.6 (3.4, 5.9) | 5.6 (5.2, 5.9) | 5.1 (4.3, 5.9) |
| Indigenous (First Nations, Inuk/Inuit, Métis) | 5.1 (4.5, 5.6) | 5.8 (4.9, 6.7) | 3.2 (3.1, 3.4) | 4.7 (4.2, 5.2) |
| Latin American | 1.5 (1.1, 1.9) | 2.7 (1.5, 3.9) ^E^ | 1.6 (1.4, 1.7) | 1.3 (1.0, 1.7) |
| Middle Eastern (Arab, West Asian) | 3.9 (3.3, 4.5) | 4.5 (3.1, 5.8) ^E^ | 2.4 (2.2, 2.6) | 1.7 (1.2, 2.1) |
| South Asian | 7.1 (6.3, 8.0) | 8.1 (6.3, 10.0) | 5.6 (5.3, 5.9) | 4.1 (3.4, 4.8) |
| Southeast Asian (Southeast Asian, Filipino) | 4.7 (4.0, 5.4) | 6.0 (4.6, 7.4) | 3.7 (3.4, 3.9) | 4.2 (3.4, 5.0) |
| White | 60.5 (59.1, 61.9) | 55.1 (52.1, 58.2) | 71.1 (70.4, 71.7) | 71.4 (69.8, 73.0) |
| Another race/ethnicity (multiple origins, Other) | 7.6 (6.8, 8.4) | 7.8 (5.9, 9.7) | 4.0 (3.8, 4.2) | 3.6 (2.9, 4.3) |
| Immigration status |  |  |  |  |
| Canadian-born | 83.6 (82.5, 84.7) | 86.5 (84.4, 88.6) | 68.9 (68.3, 69.5) | 74.1 (72.5, 75.7) |
| ≥ 10 years since immigration | 5.6 (4.8, 6.3) | 3.3 (2.1, 4.4) ^E^ | 20.6 (20.1, 21.1) | 18.0 (16.7, 19.3) |
| <10 years since immigration | 8.9 (8.0, 9.8) | 8.8 (7.0, 10.6) | 7.5 (7.2, 7.8) | 4.8 (4.1, 5.6) |
| Temporary resident | 1.9 (1.5, 2.4) | 1.4 (0.9, 2.0) ^E^ | 3.0 (2.8, 3.2) | 3.0 (2.3, 3.8) |
| Household educational attainment |  |  |  |  |
| Some high school or less | 2.9 (2.4, 3.4) | 4.6 (3.3, 5.8) | 5.0 (4.8, 5.3) | 9.1 (8.2, 10.0) |
| High school graduate | 10.7 (9.8, 11.7) | 11.8 (9.8, 13.8) | 13.5 (13.2, 13.9) | 15.7 (14.6, 16.8) |
| Some post-secondary or more | 86.4 (85.3, 87.4) | 83.6 (81.3, 85.9) | 81.5 (81.0, 81.9) | 75.2 (73.8, 76.6) |
| Home ownership |  |  |  |  |
| Own | 77.5 (76.1, 78.8) | 74.9 (72.1, 77.6) | 69.3 (68.6, 69.9) | 72.3 (70.9, 73.7) |
| Rent | 22.5 (21.2, 23.9) | 25.1 (22.4, 27.9) | 30.7 (30.1, 31.4) | 27.7 (26.3, 29.1) |
| Self-rated general health |  |  |  |  |
| Fair/poor | 3.7 (3.2, 4.3) | 3.7 (2.6, 4.8) ^E^ | 11.1 (10.8, 11.4) | 14.5 (13.6, 15.4) |
| Excellent/very good/good | 96.3 (95.7, 96.8) | 96.3 (95.2, 97.4) | 88.9 (88.6, 89.2) | 85.5 (84.6, 86.4) |
| Season |  |  |  |  |
| Spring | 25.6 (24.2, 27.0) | 20.7 (18.5, 23.0) | 25.2 (24.8, 25.7) | 25.9 (24.6, 27.1) |
| Summer | 21.5 (20.2, 22.9) | 28.6 (26.0, 31.1) | 23.5 (23.1, 23.9) | 21.9 (20.7, 23.0) |
| Fall | 25.1 (23.7, 26.5) | 25.3 (22.9, 27.7) | 25.8 (25.4, 26.3) | 24.8 (23.5, 26.0) |
| Winter | 27.8 (26.4, 29.3) | 25.4 (22.9, 27.9) | 25.5 (25.0, 25.9) | 27.5 (26.1, 29.0) |
| Community size |  |  |  |  |
| Small population centre (1,000-29,999 persons ) | 12.7 (12.0, 13.4) | 25.3 (23.5, 27.1) | 12.0 (11.5, 12.5) | 32.9 (31.1, 34.7) |
| Medium population centre (30,000-99,999 persons) | 10.6 (9.9, 11.2) | 9.0 (7.8, 10.3) | 11.3 (10.8, 11.8) | 8.0 (7.3, 8.6) |
| Large population centre (≥100,000 persons ) | 76.7 (75.8, 77.6) | 65.7 (63.5, 67.9) | 76.7 (76.2, 77.3) | 59.1 (57.3, 60.9) |
| Mean PA_TRA_, minutes/day^1^ | 30.4 (29.0, 31.8) | 26.8 (24.0, 29.7) | 16.2 (15.8, 16.6) | 15.2 (13.9, 16.6) |
| Mean PA_REC_, minutes/day^1^ | 35.6 (34.3, 36.8) | 28.5 (26.2, 30.7) | 16.2 (15.9, 16.5) | 13.4 (12.6, 14.1) |
| Area-level |  |  |  |  |
| CAN-Marg scores |  |  |  |  |
| Mean households and dwelling, score^2^ | -0.2 (-0.2, 0.2) | -0.2 (-0.2, 0.1) | 0.2 (0.2, 0.2) | 0.0 (0.0, 0.1) |
| Mean material resources, score^2^ | -0.2 (-0.2, 0.2) | 0.0 (0.0, 0.1) | -0.2 (-0.2, -0.2) | 0.0 (0.0,0.0) |
| Mean age and labour force, score^2^ | -0.4 (-0.4, 0.3) | -0.4 (-0.4, 0.3) | -0.2 (-0.2, -0.2) | -0.1 (-0.1, 0.0) |
| Mean immigration and visible minority, score^2^ | 0.5 (0.5, 0.6) | 0.6 (0.5, 0.7) | 0.4 (0.4, 0.4) | 0.3 (0.2, 0.3) |
| Mean Can-ALE index^3^ | 0.4 (0.3, 0.5) | 0.1 (0.0, 0.2) | 3.1 (3.0, 3.1) | 0.3 (0.1, 0.4) |
| Modified Can-ALE class |  |  |  |  |
| Class 1 | 16.8 (15.9, 17.7) | 27.7 (25.7, 29.7) | 14.1 (13.4, 14.8) | 34.3 (32.5, 36.0) |
| Class 2 | 40.0 (38.7, 41.3) | 30.5 (28.0, 32.9) | 37.5 (36.4, 38.7) | 27.5 (25.8, 29.1) |
| Class 3 | 30.8 (29.4, 32.1) | 29.1 (26.3, 31.9) | 29.8 (28.5, 31.1) | 23.9 (22.3, 25.5) |
| Class 4/5 | 12.4 (11.4, 13.4) | 12.7 (10.6, 14.8) | 18.6 (17.7, 19.4) | 14.4 (13.0, 15.8) |
| Mean, Normalized Difference Vegetation Index (NDVI) | 0.3 (0.3, 0.3) | 0.3 (0.3, 0.3) | 0.3 (0.3, 0.3) | 0.3 (0.3, 0.3) |

*Notes:* Data are from urban adults who participated in the 2015-2018 Canadian Community Health Survey – Annual component (n=153,708). Acronyms: n = sample size, PA_TRA_ = transportational physical activity; PA_REC_ = recreational physical activity; CAN-Marg = Canadian Marginalization Index. Presented are the weighted % (95% confidence interval), unless otherwise specified.

^1^ Daily average (minutes/day) domain-specific physical activity was calculated by dividing the weekly domain-specific physical activity by 7.

^2^ Factor scores for each of the 2016 CAN-Marg dimensions is on an asymmetrical standardized scale, with a mean of zero and standard deviation.

^3^ The Can-ALE index is the sum of the z-scores for each ALE measure (intersection density, dwelling density, points of interest, and transit measures).

^E^ The proportion estimate Is associated with high sampling based on the coefficient of variance (CV = 15.0-35.0)

**Supplemental Table 3.** Secondary analysis, reported as β (95% CI), stratifying the primary models by sex and community size among youth only in the 2015-2018 Canadian Community Health Survey (n=8,825).

| Models | Transportational  physical activity^1^ | Recreational  physical activity^1^ |
| --- | --- | --- |
|  | β (95% CI) | β (95% CI) |
| Males (n=4,619)^2^ | | |
| Main effects |  |  |
| NDVI | 0.4 (-0.28, 1.17) | 0.4 (-0.44, 1.14) |
| Can-ALE | 0.1 (0.03, 0.12) | 0.0 (-0.01, 0.08) |
| Interaction |  |  |
| NDVI × Can-ALE | 0.5 (0.22, 0.77) | 0.1 (-0.18, 0.47) |
| Females (n=4,206)^2^ | | |
| Main effects |  |  |
| NDVI | 0.5 (-0.19, 1.23) | -0.6 (-1.41, 0.27) |
| Can-ALE | 0.1 (0.01, 0.10) | -0.0 (-0.06, 0.04) |
| Interaction |  |  |
| NDVI × Can-ALE | 0.4 (-0.01, 0.77) | -0.4 (-0.80, 0.02) |
| Small community size (n=2,227)^3^ | | |
| Main effects |  |  |
| NDVI | 0.1 (-0.63, 0.91) | 0.3 (-0.52, 1.10) |
| Can-ALE | 0.3 (0.09, 0.43) | -0.1 (-0.29, 0.12) |
| Interaction |  |  |
| NDVI × Can-ALE | 1.0 (-0.15, 2.10) | 0.5 (-0.83, 1.75) |
| Medium community size (n=1,455)^3^ | | |
| Main effects |  |  |
| NDVI | 0.5 (-0.60, 1.61) | -0.3 (-1.62, 1.04) |
| Can-ALE | 0.1 (-0.06, 0.21) | -0.1 (-0.24, 0.07) |
| Interaction |  |  |
| NDVI × Can-ALE | 1.2 (0.10, 2.37) | 0.1 (-1.11, 1.26) |
| Large community size (n=5,143)^3^ | | |
| Main effects |  |  |
| NDVI | 0.5 (-0.20, 1.25) | -0.3 (-1.12, 0.54) |
| Can-ALE | 0.1 (0.03, 0.10) | 0.0 (-0.02, 0.05) |
| Interaction |  |  |
| NDVI × Can-ALE | 0.5 (0.19, 0.72) | -0.0 (-0.33, 0.23) |

*Notes:* Data are from urban adults who participated in the 2015-2018 Canadian Community Health Survey – Annual component (n=8,825). β and 95% CI are rounded to two decimal places. Acronyms: β = beta coefficient, CI = confidence interval; NDVI = Normalized Difference Vegetation Index; Can-ALE = Canadian Active Living Environments

^1^ Daily average (minutes/day) domain-specific physical activity was calculated by dividing the weekly domain-specific physical activity by 7, and then log-transforming with a natural algorithm.

^2^ Estimates are adjusted for age, Indigenous identity and race/ethnicity, immigration status, household educational attainment, home ownership, self-rated general health, season of data collection, neighbourhood households and dwelling, neighbourhood material resources, neighbourhood age and labour force, and neighbourhood immigration and visible minority.

^3^ Estimates are adjusted for sex, age, Indigenous identity and race/ethnicity, immigration status, household educational attainment, home ownership, self-rated general health, season of data collection, neighbourhood households and dwelling, neighbourhood material resources, neighbourhood age and labour force, and neighbourhood immigration and visible minority.

**Supplemental Table 4.** Secondary analysis, reported as β (95% CI), stratifying the primary models by sex, age group, and community size among adults only in the 2015-2018 Canadian Community Health Survey (n=124,102).

| Models | Transportational  physical activity^1^ | Recreational  physical acitvity^1^ |
| --- | --- | --- |
|  | β (95% CI) | β (95% CI) |
| Males (n=55,818)^2^ | | |
| **Main effects** |  |  |
| NDVI | 0.1 (-0.09, 0.38) | 0.4 (0.12, 0.62) |
| Can-ALE | 0.1 (0.07, 0.08) | 0.0 (0.00, 0.02) |
| **Interaction** |  |  |
| NDVI × Can-ALE | 0.2 (0.19, 0.29) | 0.0 (-0.01, 0.11) |
| Females (n=68,284)^2^ | | |
| **Main effects** |  |  |
| NDVI | 0.2 (0.00, 0.47) | 0.2 (0.02, 0.44) |
| Can-ALE | 0.1 (0.04, 0.08) | 0.0 (-0.01, 0.01) |
| **Interaction** |  |  |
| NDVI × Can-ALE | 0.3 (0.23, 0.42) | 0.0 (-0.04, 0.07) |
| Aged 18-39 years (n=39,043)^3^ | | |
| **Main effects** |  |  |
| NDVI | 0.2 (-0.05, 0.52) | 0.1 (-0.18, 0.38) |
| Can-ALE | 0.1 (0.05, 0.07) | 0.0 (0.00, 0.01) |
| **Interaction** |  |  |
| NDVI × Can-ALE | 0.3 (0.23, 0.37) | 0.0 (-0.03, 0.09) |
| Aged 40-64 years (n=49,951)^3^ | | |
| **Main effects** |  |  |
| NDVI | 0.2 (-0.10, 0.43) | 0.4 (0.16, 0.65) |
| Can-ALE | 0.1 (0.05, 0.09) | 0.0 (-0.01, 0.01) |
| **Interaction** |  |  |
| NDVI × Can-ALE | 0.4 (0.23, 0.47) | 0.1 (-0.01, 0.13) |
| Aged 65+ years (n=35,108)^3^ | | |
| **Main effects** |  |  |
| NDVI | -0.1 (-0.42, 0.15) | 0.5 (0.17, 0.77) |
| Can-ALE | 0.1 (0.05, 0.08) | 0.0 (-0.02, 0.01) |
| **Interaction** |  |  |
| NDVI × Can-ALE | 0.1 (-0.02, 0.16) | 0.0 (-0.07, 0.11) |
| Small community size (n=30,241)^4^ | | |
| **Main effects** |  |  |
| NDVI | -0.3 (-0.50, -0.02) | 0.0 (-0.26, 0.32) |
| Can-ALE | 0.2 (0.10, 0.21) | 0.0 (-0.06, 0.06) |
| **Interaction** |  |  |
| NDVI × Can-ALE | -0.1 (-0.43, 0.31) | 0.2 (-0.20, 0.63) |
| Medium community size (n=22,841)^4^ | | |
| **Main effects** |  |  |
| NDVI | -0.1 (-0.47, 0.23) | 0.3 (-0.05, 0.69) |
| Can-ALE | 0.1 (0.03, 0.11) | 0.0 (-0.06, 0.02) |
| **Interaction** |  |  |
| NDVI × Can-ALE | 0.3 (0.00, 0.61) | -0.2 (-0.59, 0.12) |
| Large community size (n=71,020)^4^ | | |
| **Main effects** |  |  |
| NDVI | 0.3 (0.08, 0.56) | 0.4 (0.16, 0.60) |
| Can-ALE | 0.1 (0.05, 0.07) | 0.0 (0.00, 0.01) |
| **Interaction** |  |  |
| NDVI × Can-ALE | 0.3 (0.22, 0.36) | 0.0 (-0.02, 0.07) |

*Notes:* Data are from urban adults who participated in the 2015-2018 Canadian Community Health Survey – Annual component (n=124,102). β and 95% CI are rounded to two decimal places. Acronyms: β = beta coefficient, CI = confidence interval; NDVI = Normalized Difference Vegetation Index; Can-ALE = Canadian Active Living Environments

^1^ Daily average (minutes/day) domain-specific physical activity was calculated by dividing the weekly domain-specific physical activity by 7, and then log-transforming with a natural algorithm.

^2^ Estimates are adjusted for age, Indigenous identity and race/ethnicity, immigration status, household educational attainment, home ownership, self-rated general health, season of data collection, neighbourhood households and dwelling, neighbourhood material resources, neighbourhood age and labour force, and neighbourhood immigration and visible minority.

^3^ Estimates are adjusted for sex, age (continuous), Indigenous identity and race/ethnicity, immigration status, household educational attainment, home ownership, self-rated general health, season of data collection, neighbourhood households and dwelling, neighbourhood material resources, neighbourhood age and labour force, and neighbourhood immigration and visible minority.

^4^ Estimates are adjusted for sex, age, Indigenous identity and race/ethnicity, immigration status, household educational attainment, home ownership, self-rated general health, season of data collection, neighbourhood households and dwelling, neighbourhood material resources, neighbourhood age and labour force, and neighbourhood immigration and visible minority.

**Supplemental Table 5.** Secondary analysis, reported as β (95% CI), excluding individuals with data collected during the winter months among youth and adults in the 2015-2018 Canadian Community Health Survey (n=98,979).

| Models | Transportational  physical activity^1^ | Recreational  physical activity^1^ |
| --- | --- | --- |
|  | β (95% CI) | β (95% CI) |
| Youth (n=6,362) | | |
| **Main effects** |  |  |
| NDVI | 0.6 (-0.08, 1.20) | -0.2 (-0.85, 0.54) |
| Can-ALE | 0.1 (0.02, 0.09) | 0.0 (-0.02, 0.06) |
| **Interaction** |  |  |
| NDVI × Can-ALE | 0.4 (0.18, 0.66) | -0.1 (-0.34, 0.23) |
| Adults (n=92,617) | | |
| **Main effects** |  |  |
| NDVI | 0.1 (-0.04, 0.34) | 0.3 (0.12, 0.51) |
| Can-ALE | 0.1 (0.06, 0.08) | 0.0 (-0.01, 0.01) |
| **Interaction** |  |  |
| NDVI × Can-ALE | 0.2 (0.18, 0.30) | 0.1 (0.00, 0.11) |

*Notes:* Data are from urban individuals who participated in the 2015-2018 Canadian Community Health Survey – Annual component (n=98,979). β and 95% CI are rounded to two decimal places. Estimates are adjusted for sex, age, Indigenous identity and race/ethnicity, immigration status, educational attainment, home ownership, self-rated general health, season of data collection, community size, neighbourhood households and dwelling, neighbourhood material resources, neighbourhood age and labour force, and neighbourhood immigration and visible minority. Acronyms: β = beta coefficient, CI = confidence interval; NDVI = Normalized Difference Vegetation Index; Can-ALE = Canadian Active Living Environments.

^1^ Daily average (minutes/day) domain-specific physical activity was calculated by dividing the weekly domain-specific physical activity by 7, and then log-transforming with a natural algorithm.
